# Limits to Diffusive O_2_ Transport: Flow, Form, and Function in Nudibranch Egg Masses from Temperate and Polar Regions

**DOI:** 10.1371/journal.pone.0012113

**Published:** 2010-08-11

**Authors:** Amy L. Moran, H. Arthur Woods

**Affiliations:** 1 Department of Biological Sciences, Clemson University, Clemson, South Carolina, United States of America; 2 Division of Biological Sciences, University of Montana, Missoula, Montana, United States of America; University of Queensland, Australia

## Abstract

**Background:**

Many aquatic animals enclose embryos in gelatinous masses, and these embryos rely on diffusion to supply oxygen. Mass structure plays an important role in limiting or facilitating O_2_ supply, but external factors such as temperature and photosynthesis can play important roles as well. Other external factors are less well understood.

**Methodology/Principal Findings:**

We first explored the effects of water flow on O_2_ levels inside nudibranch embryo masses and compared the effects of flow on masses from temperate and polar regions. Water flow (still vs. vigorously bubbled) had a strong effect on central O_2_ levels in all masses; in still water, masses were considerably more hypoxic than in bubbled water. This effect was stronger in temperate than in polar masses, likely due to the increased metabolic demand and O_2_ consumption of temperate masses. Second, we made what are to our knowledge the first measurements of O_2_ in invertebrate masses in the field. Consistent with laboratory experiments, O_2_ in Antarctic masses was high in masses *in situ*, suggesting that boundary-layer effects do not substantially limit O_2_ supply to polar embryos in the field.

**Conclusions/Significance:**

All else being equal, boundary layers are more likely to depress O_2_ in masses in temperate or tropical regions; thus, selection on parents to choose high-flow sites for mass deposition is likely greater in warm water. Because of the large number of variables affecting diffusive O_2_ supply to embryos in their natural environment, field observations are necessary to test hypotheses generated from laboratory experiments and mathematical modeling.

## Introduction

Many marine and aquatic animals enclose embryos in gelatinous masses, and embryos in gel depend largely or entirely on diffusion to supply O_2_ for metabolism. Egg masses, because they are simple compared to whole organisms, provide powerful systems for examining how constraints on oxygen diffusion shape the evolution of morphology, ecology, and behavior. Studies of oxygen diffusion have demonstrated that the often thin and elaborate shapes of egg masses, along with expenditures of energy on gel that spaces embryos and lowers the overall metabolic demand of masses, help embryos to avoid severe oxygen deprivation [1]–[12]. Certainly other selective factors have also shaped egg mass morphology, including desiccation, UV, and predation [13]–[16], and selection for reducing the energy and time spent finding deposition sites [17]. However, because O_2_ is fundamentally important to the metabolism that supports development, diffusive O_2_ supply is likely an overriding factor constraining the range of possible mass morphologies.

For marine embryo masses, external oxygen environments vary over many scales [18] and the result of this variation is likely to be intensification, or in some cases relaxation, of oxygen-related constraints on morphology. Small-scale (mm–cm) variation is driven by the respiration and photosynthesis of other nearby plants and microorganisms [4], [11], [19]–[21], and in these environments embryos are exposed to fluctuating O_2_ that varies temporally on scales of minutes to days [4], [19]–[21]. Meso-scale variation (m–km) is driven by the mixing of pools of differentially-oxygenated water [18]. On a global scale, water O_2_ content is predictably high in some parts of the oceans (e.g., the nearshore and deep Southern Ocean) and low in others (e.g., the tropics, deep sea). In hypoxic environments, embryos are exposed to substantially lower O_2_ levels during development [5], [11]. Models and experiments have tended to focus on mass structure, e.g. shape, size, and density of embryos, and have rarely considered the effects of external factors such as ambient O_2_ concentration on O_2_ flux to embryos.

Rate of O_2_ supply to embryos in masses depends not only on ambient O_2_ levels, but also on how fast O_2_ is replenished at the surface of the mass. When boundary layers are large, as in stagnant water, embryos in egg masses deplete O_2_ not only in the egg mass itself but also in its boundary layer, thus increasing the distance over which O_2_ must diffuse [18], [22], [23]. In rapidly moving water, by contrast, the oxygen-depleted layer around the egg mass is constantly refreshed and diffusion distances are shorter. Boundary layers are ubiquitous and likely affect internal O_2_ in invertebrate masses [1], [5], [11]; in large, non-gelatinous fish embryo masses, development is only supported in regions of high (10–15 cm/s) flow where internal O_2_ is maintained above 70% [24].

All else being equal, however, flow should impact O_2_ in masses more when metabolic demand of the masses is high, meaning embryos deplete O_2_ more rapidly–for example, in regions with warmer water [10]. Likewise, flow should have a greater impact on O_2_ supply in masses in ambient hypoxia. One broad prediction, then, is that compared to masses from cold, highly-oxygenated regions like the polar oceans, temperate and tropical masses, which tend to inhabit warmer and less oxygenated parts of the ocean, likely experience both greater overall hypoxia and stronger selection for behavioral, physiological, or morphological adaptations to reduce the effects of boundary layers. The converse should also be true; in cold, well-oxygenated high-latitude oceans, embryos in masses should experience higher O_2_ overall; likewise, the effect of water flow on O_2_ supply may be reduced compared to lower latitudes.

Our goals were (1) to determine how external water flow affects internal O_2_ concentrations in egg masses in the laboratory, (2) to compare the effects of flow among species from temperate regions and from the Southern Ocean surrounding Antarctica; and (3) to measure internal O_2_ concentrations of marine egg masses *in situ*, and to compare laboratory and field data to assess the utility of models for predicting natural field O_2_ conditions for embryos. Studies of amphibian egg masses have shown that processes affecting O_2_ supply and demand are complex and that simple models generally do not capture O_2_ profiles in nature [7]. Oxygen profiles in marine egg masses are logistically difficult to measure and, to our knowledge, have never been performed for invertebrates.

## Methods

### Study organisms

We studied gastropods in the order Nudibranchia, which are a diverse (>3,000 named species) group of molluscs that are shell-less and largely predatory as adults and have a worldwide distribution [25]. Nudibranchs lay benthic, gelatinous egg masses containing hundreds to hundreds of thousands of embryos [26]. In some taxa, offspring hatch as free-living, swimming veliger larvae; others lack a planktonic larval stage and hatch as metamorphosed juveniles [27], [28]. The dynamics of O_2_ in nudibranch embryo masses have been well studied in the laboratory in the context of morphological traits like mass size and shape, and density of embryos [e.g. [1], [3]–[5]], and the interactive effects between these characters and temperature [10]–[12].

### Specimen collection

Egg masses and adult nudibranchs were collected at two sites: Friday Harbor, Washington, in the cold-temperate eastern Pacific ( = NE Pacific), where collections were made from floating docks, intertidal habitats, and on SCUBA; and McMurdo Sound, Antarctica ( =  Antarctica) where collections were made on SCUBA. Animals and masses were brought back to the laboratory and kept in mesh-walled cages in running sea water at ambient or near-ambient temperatures (12°C and −1°C, respectively). Adults were kept in species-specific groups to allow identification of egg masses, and adult cages were checked daily for new egg masses. Egg masses were obtained mostly from adults kept in the laboratory, but in some cases (particularly for Antarctic taxa, which took considerably longer to lay egg masses in the laboratory) masses were collected in the field by SCUBA divers. When possible, field-collected egg masses were identified morphologically; in some cases, particularly among the poorly-known Antarctic taxa, we relied on mtCOI sequencing to place egg masses on a phylogeny (Shields C, Marko PB, Woods HA, and Moran AL, in prep.). Diameters of egg masses were measured with calipers; for egg masses that were sheet-like rather than cylindrical, we measured ‘diameter’ as the thickness of the sheet.

### Oxygen concentrations under high and low flow in the laboratory

Radial profiles of oxygen in egg masses were measured using Clark-style microelectrodes (50 µm tips; Unisense, Denmark) connected to a picoammeter (PA2000, Unisense). Analog outputs from the picoammeter were sent to an A/D converter (UI2, Sable Systems, Las Vegas, NV) and recorded onto a computer using Expedata software (v. 1.0.17, Sable Systems). Electrodes were calibrated frequently (several times per day, usually before every radial profile) using N_2_-bubbled seawater as the zero and air-bubbled seawater as the span, always at the same water temperature in which measurements would subsequently be made. Stable water temperatures (12°C and −1°C) were obtained using a water-jacketed set of cells, one for calibration (20 ml volume) and the other (70 ml) for measurements of egg masses. Both cells were supplied with compressed gases, which were directed through sintered glass (providing a stream of small-diameter bubbles) and needle valves for flow control. Water or a water-alcohol solution was recirculated through the water jacket from a constant-temperature bath. During measurements, water temperatures in the measurement cell were logged continuously using a calibrated type-T thermocouple connected to a meter (TC-1000, Sable Systems); in general, temperature deviated <0.5°C from the water bath's set point.

To determine how strongly water flow affected internal O_2_ concentrations in egg masses, we measured central O_2_ levels with and without external stirring. Rapid stirring of seawater (giving small boundary layers around egg masses) was achieved by bubbling air vigorously into the cell. Water flow rates were not measured directly, but drops of dye introduced into stirred water were completely dispersed (into the 70-ml volume) within 5 s. Unstirred seawater was obtained by turning off the air stream. The water was of course not completely still, as small temperature gradients led to some convective flow; however, flow rates were very low (and hence boundary layers around the egg mass very large); drops of dye put into still water took many minutes to disperse.

In a typical experiment, an egg mass was first submerged in the measurement cell and pinned loosely to a platform of Nitex mesh [see 11, 20]. The experimental cell was then bubbled with air, to give well-stirred, air-saturated seawater around the mass. Meanwhile, the electrode was calibrated (in the calibration cell) and then moved to the experimental cell. There was no shift in the electrode calibration when the electrode was exposed to air for a few seconds during transfer. We then measured the oxygen content of the seawater around the egg mass, after which we advanced the electrode tip with the use of a micromanipulator (viewed through a stereomicroscope) into the center of the pinned egg mass. The electrode was left in the center for up to several hours, until central oxygen levels appeared to reach a stable level. Equilibration times were short (<30 min) in temperate egg masses and longer (several hours) in Antarctic masses, reflecting the relative rates of metabolism and oxygen transport. Subsequently, the stream of air was turned off, giving still water in the measurement cell. Central O_2_ levels were again monitored until a new, lower equilibrium level of oxygen was reached.

### Oxygen concentrations in the field

O_2_ concentrations were measured in egg masses in the field by SCUBA divers using an underwater picoammeter connected to a Clark-style microelectrode (50 µm tip, UWMeter, Unisense, Inc; Denmark). Measurements were made on both natural egg masses *in situ*, and on artificial egg masses that were outplanted in the field 24 h previously (methods for constructing and out-planting artificial egg masses are described below). All measurements from artificial masses were made in Friday Harbor, Washington, at a depth of 5 m; readings of natural egg masses were made near McMurdo Station, Antarctica, at depths of between 15 and 30 m. Prior to dives, the meter and probe were calibrated at the surface with air- and nitrogen-bubbled water at the working temperature (12°C, NE Pacific; −1.8°C, Antarctica). The meter and probe were connected by a ∼2 m cord, which allowed one diver to hold the meter and record data while another manipulated the probe. Once an egg mass was located, three baseline O_2_ measurements were taken in the water column approximately 1 m above the mass. The probe was next positioned just above and almost touching the mass, and three readings were taken. Finally, the tip of the probe was pushed by hand slowly through the egg mass until it was at the center, at which point the measured O_2_ level was recorded. The probe was then removed from the mass and the final step was repeated twice, for a total of three replicate internal measurements for each mass. The mean of the three water column and the three surface readings were used for analyses. For internal readings, instead of the mean we used the lowest reading because it was most likely to have been taken at the center of the mass (positioning the probe in the mass was substantially more difficult in the field than in the laboratory).

Field measurements were made primarily on egg masses from the Antarctic species *Tritonia challengeriana* Bergh 1884 and *Tritoniella belli* Elliot 1907. Measurements were also made on the masses of two other Antarctic species which we could not identify by morphology: “beige” masses and “barrel” masses (both of which were >20% divergent from any other Antarctic sequence at COI (Shields C, Marko PB, Woods HA, and Moran AL in prep.).

After O_2_ readings were obtained, masses were collected by divers and transported on ice back to McMurdo Station and maintained in running seawater at −1°C. The diameter of each egg mass was measured with calipers. For each field-measured egg mass, we measured internal O_2_ concentrations in the lab under still and bubbled conditions as described above. Subsequently, embryos from each mass were photographed under a calibrated compound microscope and their developmental stages determined. Embryos were categorized into eight stages; (1) early cleavage (2–16 cell), (2) morula, (3) blastula - gastrula, (4) trochophore, (5) early veliger (very thick velar lobes, distinct foot, no shell), (6) mid veliger (shell, larger and thinner velar lobes, fully formed foot, cap shell), (7) late veliger (statocysts, eyespots, fully formed velum, full shell) and (8) hatching (velum reduced in size or absent, foot fully formed and used for crawling; when live animals were observed under the microscope, most were crawling on the foot or were withdrawn entirely into shells). For some analyses, embryos were classified only as pre- or post-veliger. Samples of each egg mass were preserved in 95% ethanol for later molecular analysis to confirm identity (Shields C, Marko PB, Woods HA, and Moran AL in prep.). Species, collection localities, and numbers of masses used in experiments are given in Table 1.

**Table 1:** List of species, source locations, and diameters of masses used in linear mixed effects (LME) models.

		Species	Location	Number of masses*	Average diameter (mm) ± SE
San Juan Islands, WA, USA		*Aeolidia papillosa*	Friday Harbor; Argyle Creek	2 (2)	0.75±0.09
(NE Pacific)		*Dendronotus frondosus*	Friday Harbor; lab docks	2 (0)	--
		*Dirona albolineata*	Friday Harbor; lab docks and subtidal	1 (0)	--
		*Dialula sandiegensis*	Friday Harbor; lab docks	2 (2)	0.80±0.05
		*Doto columbiana*	Friday Harbor; Shaw Island subtidal	5 (4)	0.82±0.03
		*Eubranchus sanjuanensis*	Friday Harbor; Shady Cove subtidal	4 (3)	0.57±0.09
		*Geitodoris heathi*	Friday Harbor; lab docks	2 (1)	0.87
		*Melibe leonina*	Friday Harbor; Lopez Island	7 (3)	1.1±0.06
		*Rostanga pulchra*	Friday Harbor intertidal	2 (2)	0.72±0.12
		*Triopha maculata*	Friday Harbor docks and subtidal	7 (3)	1.26±0.16
		*Tritonia diomedea*	Friday Harbor subtidal	1 (0)	--
McMurdo Sound, Antarctica		*Doto antarctica*	McMurdo Sound subtidal	8 (8)	1.75±0.10
		*Tritonia challengeriana*	McMurdo Sound subtidal	9 (4)	3.00±0.21
		*Tritoniella belli*	McMurdo Sound subtidal	18 (17)	4.50±0.14
		“Barrel” egg masses	McMurdo Sound subtidal	3 (1)	0.71
		“Beige” egg masses	McMurdo Sound subtidal	6 (5)	3.88±0.32

*Number in parentheses is the number of masses for which diameters were available.

### Artificial egg masses

To compare field and lab measurements in a controlled system, artificial agarose masses containing early embryos of *Dendraster excentricus* were created from low-melting point agarose gel after the methods of Strathmann and coworkers [3], [5], [29], as modified by the authors [10] with some further modifications described here, and deployed in the field.

Adult *D. excentricus* were collected in Puget Sound, Washington and maintained in running seawater. Adults were spawned and gametes fertilized following published procedures [26]. Newly fertilized embryos were allowed to proceed through the second cleavage stage to establish that cultures were developing normally. To make artificial egg masses, a 2% solution of agarose and seawater was cooled with stirring to 30°C. 10 ml of a known concentration of embryos at the 2–4-cell stage of development was added to 20·ml of 2% agarose solution to make a solution at a final concentration of 1.5% agarose and 6 embryos µl^−1^. Control masses were made by mixing 20 ml of 2% agarose with 10 ml of plain seawater filtered to 0.2 µm. Agarose solutions were immediately poured into cylindrical moulds (individual wells of a polypropylene microcentrifuge tube rack, 11 mm diameter x 30 mm depth (Fisher Scientific, Catalog #05–541), immersed in 12°C water (ambient) in a sea table, and allowed to set for 10 minutes.

For deployment in the field, 6 experimental and 6 control egg masses were attached to a 2-m piece of PVC pipe with holes drilled at 10-cm intervals. Masses were attached to the rack by 10–15 cm lengths of monofilament line that were tied to the rack at one end and tied to small plastic disks embedded in each mass at the other (discs and line had been placed in each mold prior to adding melted agarose). To avoid potential positional effects, experimental and control masses were attached in alternating order along the pipe.

The PVC rack was then placed in a container of sea water and transported to the field site. Divers on SCUBA placed the array between two pilings under the docks at the Friday Harbor Laboratories at 5 m depth and approximately 1 m off the bottom (to avoid silt and benthic predators). After 24 h, *in situ* measurements of O_2_ concentrations in the masses were taken as described for field masses in the Antarctic with two modifications: we took two (instead of three) measurements at the surface of each mass, and four separate measurements of central O_2_ concentrations. After field measurement, the masses were returned to the laboratory and internal O_2_ was measured for each mass under fully oxygenated and still-water conditions as described for natural masses. Embryos were at the unhatched blastula stage after the field outplants, and field and laboratory measurements on artificial masses were performed at ∼12°C.

### Statistical Analyses

Because individual egg masses were measured multiple times, we used linear mixed effects (LME) models [30] to analyze the data. In general, individual masses were nested under species, with mass identity as a random effect (R statistical package, v 2.8.1). Fixed effects included Flow (bubbled vs. still), Location (Antarctic vs. NE Pacific), Stage (preveliger vs. veliger-to-hatching), and Diameter. We first used all data to test for effects of Location and Flow (without including Stage or Diameter as factors). Then, because data on either Stage or Diameter were missing for some masses, we performed analyses testing each factor separately, which maximized our ability to detect single-factor effects if present.

To analyze field measurements of natural egg masses, we used the two species for which we had the largest data set, *Tritoniella belli* (25 masses) and *Tritonia challengeriana* (12 masses). We performed a multiple regression using field internal O_2_ as the dependent variable and Diameter and Stage as independent variables. All linear regressions were performed in Systat 11.0 (Systat, Inc., Chicago, IL). To analyze field measurements of artificial masses, we used Student's t-tests comparing external and central O_2_ concentrations of experimental and control masses. For statistical comparisons, we used the average of two measurements taken just above the surface of each mass and the lowest of the four internal measurements taken on each mass. To determine whether field O_2_ measurements were more similar to bubbled (high-flow, O_2_ saturated conditions) or still water, we used separate paired t-tests to compare the reduction in internal O_2_ in individual masses in the field to the same masses equilibrated in (1) still water, and (2) bubbled water in the laboratory.

## Results

### Oxygen concentrations under high and low flow in the laboratory

**Figure 1:**
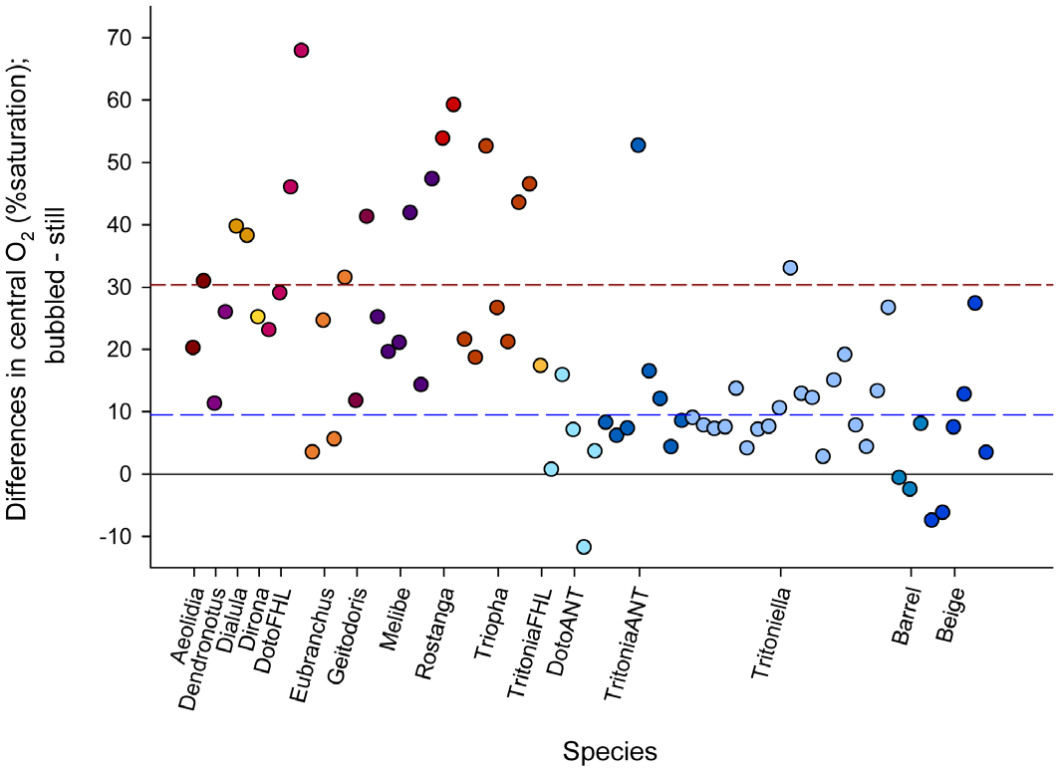
Effects of bubbling on central O_2_ in temperate vs. Antarctic masses. Each point represents the difference between central O_2_ equilibrated under bubbled, air saturated conditions, and central O_2_ measured in the same mass equilibrated in still water. The solid black line is the line of no difference; almost all masses, therefore, had higher central O_2_ under bubbled conditions than in still water. The blue dotted line is the mean difference for Antarctic masses, which is ∼20% lower than the mean difference for temperate masses (red dotted line).

Masses from both locations (NE Pacific, Antarctica) had significantly higher central O_2_ in bubbled than in still water (Table 2). Location had a marginally nonsignificant main effect on central O_2_ (higher in the Antarctic than in the NE Pacific), but the interaction between Location and Flow was highly significant (Table 2), reflecting that temperate egg masses showed much larger declines in central oxygen in still water than in bubbled (Fig. 1). Developmental Stage had a strong main effect, with Stage 2 masses (veliger or beyond) having significantly lower O_2_ than Stage 1 masses (preveliger) (Table 3); the interaction between Stage and Location was significant, though it accounted for less of the total variance. Diameter did not have a significant main effect on central O_2_ (Table 4), nor a significant interaction with any other factor. Again, however, there was a significant effect of Flow and a significant Location x Flow interaction.

**Table 2:** Summary of LME results showing effects of bubbling and location on central O_2_ in gelatinous egg masses of nudibranchs.

Source	df	denDF	F	P
Intercept	1	72	1007.1	<0.0001
Location (L)	1	14	3.6	0.08
Flow (F)	1	72	165	<0.0001
L×F	1	72	43.6	<0.0001

**Table 3:** Summary of LME results showing effects of developmental stage on central O_2_.

Source	df	denDF	F	P
Intercept	1	57	1048.1	<0.0001
Location (L)	1	11	6.3	0.028
Flow (F)	1	57	177.4	<.0001
Stage (S)	1	46	30.8	<0.0001
L×F	1	57	56.4	<0.0001
L×S	1	46	14.1	0.0005
F×S	1	57	8.8	0.0045
L×F×S	1	57	10.1	0.0024

**Table 4:** Summary of LME results showing effects of mass diameter on central O_2_.

Source	df	denDF	F	P
Intercept	1	49	486.6	<0.0001
Location (L)	1	11	0.3	0.592
Flow (F)	1	49	111.1	<0.0001
Diameter (D)	1	38	0.4	0.509
L×F	1	49	35.7	<0.0001
L×D	1	38	2.1	0.155
F×D	1	49	0.1	0.764
L×F×D	1	49	0.02	0.896

### Oxygen concentrations in the field

**Figure 2:**
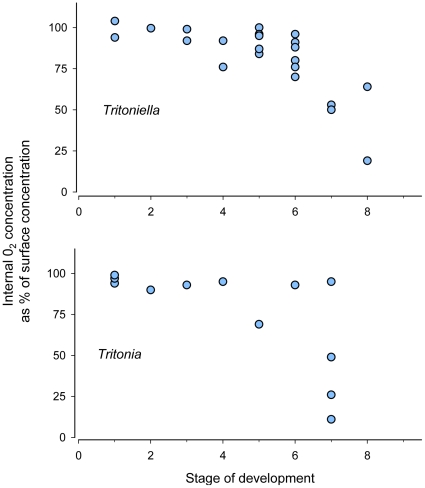
Developmental stage vs. internal O_2_. Relationship between developmental stage (x axis) and internal O_2_ concentration (y axis) for masses of two Antarctic nudibranch species, *Tritoniella belli* (top; linear regression, R^2^ = 0.46; p = <0.001) and *Tritonia challengeriana* (bottom; R^2^ = 0.39, p = 0.018).

On average, central O_2_ concentration in the field was 84.3%±3.8 (se) for *Tritoniella* (n = 27) and 75.8±8.8 (se) for *Tritonia* (n = 12). For *Tritoniella*, the species for which we had the most data (central O_2_ measurements and stage data on 25 field masses and diameter data for 21), central O_2_ was significantly affected by Stage, which explained 46% of the variance (linear regression; adjusted multiple R^2^ = 0.457; P = <0.001, N = 24 after one mass was discarded as an outlier) (Fig. 2). Central O_2_ was also significantly affected by Diameter, though it had less explanatory power than Stage (linear regression; adjusted multiple R^2^ = 0.196, p = 0.043, n = 21). Together, Stage and Diameter explained 64% of the variance in central O_2_ (multiple regression; adjusted multiple R^2^ = 0.639, p<0.0001, n = 21) for this species. For *Tritonia*, central O_2_ was also significantly affected by stage (linear regression: adj. multiple R^2^ = 0.39, p = 0.02, n = 12) (Fig. 2). Diameters were available for only three *Tritonia* masses so the effect of Diameter was not tested in this species.

**Figure 3:**
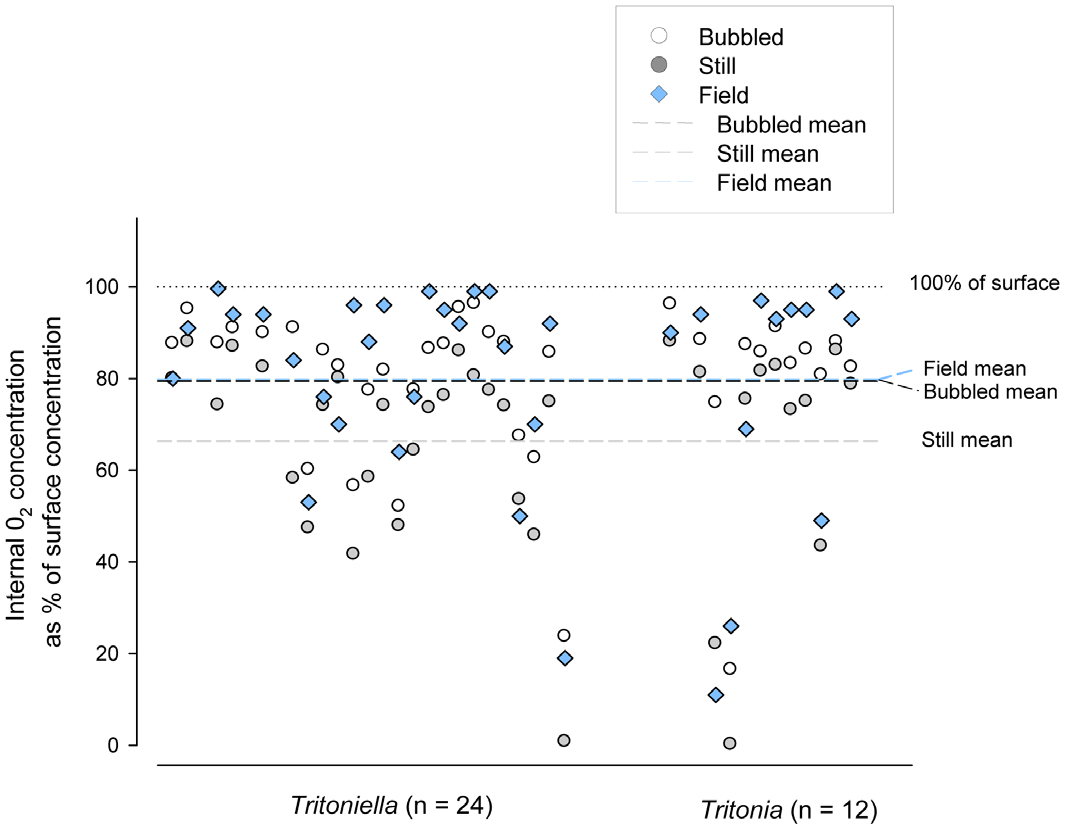
Internal O_2_ in masses in the field and the lab. Internal O_2_ in individual masses of two Antarctic nudibranch species, *Tritonia challengeriana* and *Tritoniella belli*, measured under three conditions; in the field (blue diamonds), equilibrated in the lab under bubbled conditions (open circles), and in the lab in still water (filled circles). The combined means of all field (blue dashed line, x = 79.9% of ambient), lab bubbled (black dashed line, x = 79.5%), and lab still water (grey dashed line, x = 66.3% of ambient) measurements for both species are also shown.

When field data from *Tritonia* and *Tritoniella* masses were compared with laboratory measurements of the same masses held at −1°C in still water, O_2_ levels were significantly higher for both species in the field (*Tritonia*: paired t-test on arcsine-transformed % saturation data, t = 3.33, df = 11, P = 0.007; *Tritoniella*: t = 5.8, df = 23, P<0.001). Field O_2_ measurements were not statistically distinguishable from central O_2_ under bubbled laboratory conditions for either species (Fig. 3) (*Tritonia*: t = −0.17, DF = 11, P = 0.87; *Tritoniella*, t = 1.6, DF = 23, P = 0.113).

### Artificial egg masses

**Figure 4:**
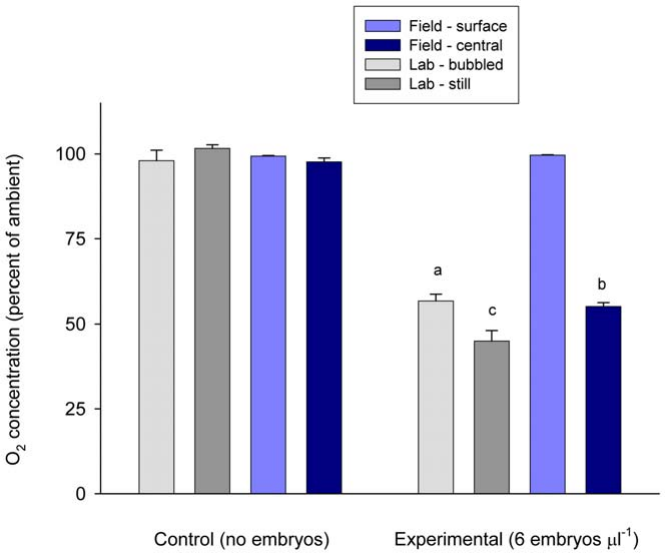
Internal O_2_ of artificial masses in the lab and field. Internal O_2_ measured in the lab (grey bars) and field (blue bars) for agarose masses containing no embryos (control; left) or containing embryos of *Dendraster excentricus* at 6 embryos ml^−1^ (experimental; right), expressed as % concentration relative to the water column. For lab masses, light grey bars are measurements taken of masses calibrated in air-bubbled water; dark grey bars represent still water conditions. For field masses the light blue bars represent readings taken at the surface of the mass and dark blue bars are readings from the center. Letters (a, b, c) above bars indicate significant differences between means. Each bar represents the mean measurements taken from four (experimental) or five (control) masses.

Of 10 artificial egg masses we outplanted, 9 were undamaged after 24 hours (5 control masses and 4 experimental masses). Central O_2_ was significantly lower in the field in masses containing embryos than in control masses (unpaired t-test, t = 28.0, DF = 7, p<0.0001) (Fig. 4). In control masses, there was no detectable drawdown of central O_2_ (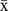 surface = 99.3%±0.2 (se) of ambient; 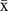 central = 97.7%±1.1 (se) of ambient) (Fig. 4), paired t-test, t = 1.5, DF = 4, P = 0.21). In contrast, in experimental masses containing embryos, central O_2_ was substantially lower than surface readings (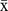 surface = 99.7%±0.2 SE of ambient; 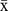 central = 55.1%±1.0 of ambient) and these differences were significant (paired t-test, t = 44.9, DF = 3, P<0.0001) (Fig. 4).

When field-deployed artificial masses were measured in the lab, control masses still showed no drawdown under either bubbled or still conditions (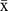 central  = 98.0%±3.1 and 101.6%±1.1 of saturation, respectively) (Fig. 4). Central O_2_ in experimental masses showed drawdown under all treatments, and was higher under bubbled conditions in the lab than either under still laboratory conditions or in the field (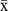 central  = 56.8%±2.0, 45.0%±3.1, and 55.1%±1.1 of saturation, respectively; one-tailed paired t-test, t = 2.7, df = 6, P = 0.036; two-tailed paired t-test, t = 28.0, df = 3, P<0.001). Central O_2_ was significantly higher in field-deployed masses than under still water in the laboratory (two-tailed paired t-test, t = −14.3, p<0.001, DF = 3) (Fig. 4).

## Discussion

Our data indicate that boundary layers strongly affect oxygen supply to embryos in gelatinous egg masses. In nearly all natural masses measured in the laboratory, central O_2_ was higher when surrounding water was vigorously bubbled (Fig. 1); in still water, average central O_2_ was 30% lower (than in flow) for NE Pacific masses and 10% lower in Antarctic masses. The magnitude of this effect, however, differed among masses, indicating that additional factors drive the relative importance of Flow. The first was Stage; masses containing developmentally advanced embryos experienced relatively greater drops in central O_2_ (Table 2), and in still water the centers of mature masses often had areas of complete anoxia. The effect of larger boundary layers is likely greater for older embryos because their metabolic demand for O_2_ is higher [10], [12]. High metabolic demand rapidly depletes O_2_ in the still water surrounding masses, and diffusion becomes increasingly insufficient for delivering O_2_ to the egg-mass surface. Constraints on mass size, shape, and structure thus act most strongly during more advanced stages of development; younger masses will likely appear to be ‘overconstructed’ (thicker, larger, with embryos at a lower density) relative to the oxygen requirements of embryos.

The second interacting factor was Location. Overall, Antarctic masses experienced substantially less O_2_ drawdown than masses from the NE Pacific; average central O_2_ in the Antarctic even in still water was >60% of saturation. Moreover, the effect of Flow was substantially (and significantly) smaller in Antarctic than in NE Pacific masses (Fig. 1, Table 3). This pattern likely reflects the effects of temperature on both sides of the supply-demand relationship for oxygen. On the supply side, oxygen movement into egg masses depends on both the diffusion coefficient and solubility of oxygen. Our direct measurements of diffusion coefficients of oxygen through egg mass jelly suggest they are relatively insensitive to temperature [11]. Oxygen solubility is also higher in the Antarctic. Together (in Krogh's constant), therefore, oxygen supply should be somewhat higher in Antarctic egg masses. On the demand side, however, metabolic consumption of oxygen is radically depressed by cold Antarctic temperatures [11], [12], [31]–[36]. Therefore, the small effects of Flow and Stage in Antarctic masses most likely reflect the lower metabolic activity of nudibranch embryos (and ectotherms in general) in extreme cold [11], [12], [31]–[36].

Although models and prior experiments suggest that mass Diameter influences oxygen profiles [3], [5], [10]–[12], [29], we found no significant effect of Diameter in laboratory studies (Table 4), either as a main effect or in interaction with other factors (Table 4). In contrast, when we compared field internal O_2_ concentrations within a single species (*Tritoniella belli*), Diameter did explain a significant, though small (∼20%), proportion of the variance in central O_2_ concentration. The effects of mass Diameter were likely detectable in field masses because these were within-species comparisons with a comparatively large (n = 21) dataset. In our laboratory experiments, low intraspecific size variability and variation in other factors (age, embryo density, physical state of the mass, variation in fouling among masses) probably obscured underlying relationships between size and central O_2_.

In nature, flow is complex and difficult to measure. Even if we could measure flow over temporal and spatial scales relevant to developing egg masses, modeling its effects on oxygen levels would be daunting. How, then, to determine what flow is like around small egg masses in nature? We examined this question by comparing central O_2_ in artificial egg masses in the field to measurements on the same masses under still and stirred conditions in the laboratory. Measurements made in stirred conditions more closely resembled field measurements (Figs. 3 and 4), suggesting that boundary layers did not limit O_2_ availability in the field in the Antarctic. We predict that the magnitude of the boundary-layer effect in nature will vary considerably depending on water temperature, mass size, embryo density, embryo age, and placement of the mass. Because local placement of masses is likely to profoundly affect O_2_ supply to embryos, adults, especially adults in temperate and tropical waters, may experience selection for depositing masses in high-flow conditions; the importance of site choice relative to flow has been demonstrated in fish that lay very large, non-gelatinous masses [24]. Anecdotally, we note that at Friday Harbor many species lay masses on the tips of hydroids, which lifts them out of the benthic boundary layer and away from other respiring organisms on the benthos.

Our data from egg masses measured *in situ* suggest that Antarctic masses are rarely O_2_ –limited in nature. Central O_2_ levels of these masses were high overall, dropping below 50% saturation in only the oldest, largest masses (Fig. 2). Likewise, in individual masses, central O_2_ levels in field masses were more similar to O_2_ levels in well-stirred, laboratory conditions than to still-water conditions (Fig. 3). High oxygenation of Antarctic egg masses likely stems from a combination of low metabolic demand (driven by low metabolism of embryos and, potentially, reduced density of embryos [11]), comparatively high ratios of oxygen supply to demand [11], [12], and the well- mixed water around McMurdo Station.

The combination of high O_2_ availability and low demand among Antarctic marine ectotherms has been implicated in a number of unique adaptations, including polar gigantism –in which diverse polar ectotherms reach much larger sizes than their relatives in warmer waters [37], [38]. This phenomenon has been attributed to a release from constraints on form and function driven by the high ratio of O_2_ supply to metabolic demand for these organisms [37], [38]; [ but see 39]. Our data from Antarctic masses suggest that it is not simply the direct effects of temperature-oxygen interactions that make the O_2_ environment unusually benign; cold-water organisms will also be less affected by low O_2_ supply from inadequate water flow and by developmental increases in metabolic rate. Thus, both deposition sites and egg mass morphologies of Antarctic species may be less constrained. In addition, because embryos in Antarctic masses rarely experience hypoxia, embryos may lose physiological mechanisms for tolerating it; this hypothesis has yet to be tested for most invertebrates.

Understanding how multiple factors interact to shape oxygen profiles in masses is a key to understanding ecological and evolutionary forces that drive interspecific and latitudinal differences in egg mass form. Comparisons of related taxa in the Antarctic and in warmer waters could provide a powerful means of testing the idea that the impact of boundary-layer limitations to diffusive O_2_ transport – and corresponding selection for physiological, physical, or behavioral traits to minimize these limitations – vary on large scales, across global clines in temperature and O_2_ availability.

Two conclusions about egg masses are increasingly clear. The first is relatively straightforward; oxygen levels in egg masses can vary profoundly in space and time, and this variation has large effects on the timing and success of embryonic development [1]–[7], [9], [10]–[12], [19], [20], [24], [29]. The second conclusion involves more complexity; multiple physical and biological factors, including (but not limited to) mass size and shape, embryo size, spacing, and age, species-specific embryonic metabolic rates, local placement, local and regional flow regimes, local and regional ambient O_2_, temperature, and biotic environment all interact to determine the actual shapes of oxygen profiles at any particular place and time. The present study adds another layer of complexity by showing that the importance of one major factor, flow, depends on several of other these other factors. Collectively, these interactions complicate the task of developing predictive models capable of mapping egg mass form and function onto ecological and evolutionary processes of interest, and highlight the importance of field measurements for establishing the oxygen environments that embryos experience in nature.
